# Extended Analysis of the Hospitalization Cost and Economic Burden of COVID-19 in Romania

**DOI:** 10.3390/healthcare13090982

**Published:** 2025-04-24

**Authors:** Alíz Bradács, László Lorenzovici, László-István Bába, Zoltán Kaló, Szabolcs Farkas-Ráduly, Andreea Mihaela Precup, Klementina Somodi, Maria Gheorghe, Alexandru Calcan, Gyöngyi Tar, Ovidiu Adam, Violeta Tincuta Briciu, Simin Aysel Florescu, Edith Simona Ianoși, Ovidiu Gârbovan, Dimitrie Cristian Siriopol, Zoltán Vokó

**Affiliations:** 1 Faculty of Medicine and Pharmacy, University of Oradea, 410087 Oradea, Romania; bradacs.aliz@yahoo.com; 2 “Dr. Mircea Pop” City Hospital Marghita, 415300 Marghita, Romania; 3 Syreon Research Romania, 540004 Tîrgu Mureș, Romania; laszlo.baba@syreon.ro (L.-I.B.); farkas_raduly_szabolcs@syreon.ro (S.F.-R.); klementina@syreon.ro (K.S.); 4 Faculty of Technical and Human Sciences, Sapientia Hungarian University of Transylvania, 540485 Tîrgu Mureș, Romania; 5 Department of Doctoral Studies, G. E. Palade University of Medicine, Pharmacy, Science and Technology, 540142 Tîrgu Mureș, Romania; csutak@nextra.ro (G.T.); ianosi_edith70@yahoo.com (E.S.I.); ovidiugirbovan@yahoo.com (O.G.); 6 Center for Health Technology Assessment, Semmelweis University, 1085 Budapest, Hungary; zoltan.kalo@syreon.eu (Z.K.); zoltan.voko@syreon.eu (Z.V.); 7 Syreon Research Institute, 1145 Budapest, Hungary; 8 Hospital Consulting, 540004 Tîrgu Mureș, Romania; andreea.precup@syreon.ro; 9 Pfizer Romania, 013686 Bucharest, Romania; maria.gheorghe@pfizer.com (M.G.); alexandru.calcan@pfizer.com (A.C.); 10 Faculty of General Medicine, Pediatric Orthopedics Department, “Victor Babeș” University of Medicine and Pharmacy Timișoara, 300041 Timișoara, Romania; adamovidiu29@yahoo.com; 11 “Louis Țurcanu” Emergency Children’s Hospital, 300011 Timișoara, Romania; 12 Department of Infectious Diseases, “Iuliu Hațieganu” University of Medicine and Pharmacy Cluj-Napoca, 400347 Cluj-Napoca, Romania; briciu.tincuta@umfcluj.ro; 13 Clinical Hospital of Infectious Diseases Cluj-Napoca, 400003 Cluj-Napoca, Romania; 14 Faculty of Medicine, Department of Infectious Diseases, University of Medicine and Pharmacy Carol Davila Bucharest, 050474 Bucharest, Romania; siminflorescu@yahoo.com; 15 “Dr. Victor Babeș” Clinical Hospital of Infectious and Tropical Diseases Bucharest, 030303 Bucharest, Romania; 16 Clinical County Hospital Tîrgu Mureș, 540136 Tîrgu Mureș, Romania; 17 Department of Nephrology, “Ștefan cel Mare” University of Suceava, 720229 Suceava, Romania; dimitrie.siriopol@yahoo.com; 18 County Emergency Hospital Suceava, 720224 Suceava, Romania

**Keywords:** burden of disease, COVID-19, hospital cost, indirect economic costs

## Abstract

**Background/Objectives:** COVID-19 has impacted Romania’s healthcare, economy, society, and public health. This study aims to evaluate the financial impact of the COVID-19 pandemic in Romania by analyzing both hospital costs and key elements of economic costs. The assessment was conducted from the perspective of the national payer. Hospital costs were analyzed covering two distinct timeframes: Q4 2020–Q3 2021 and Q1 2022–Q4 2022. The estimation of economic costs covered Q4 2020–Q3 2021. **Methods:** Hospital care costs were estimated using financial data from eight hospitals. The costs were extrapolated to inpatient data from 60 public hospitals for each of the two study periods. The disease burden was determined based on official data, including the number of confirmed cases, hospital bed occupancy, reported fatalities, and various cost components from an economic perspective. **Results:** The findings indicate that the average hospital cost per patient episode was EUR 2267 (95% CI: 2137–2396) during the first period and EUR 2003 (95% CI: 1799–2207) in the second. The total national hospitalization expenses amounted to EUR 1.35 billion and EUR 730 million, respectively. When accounting for productivity losses and testing costs, the overall expenditure reached EUR 5.39 billion for Q4 2020–Q3 2021. **Conclusions:** In conclusion, the total economic burden of the COVID-19 pandemic in Romania by the end of 2021 was estimated at EUR 5.39 billion, encompassing hospitalization, isolation, premature deaths, quarantine, testing, and parental allowances. Despite the emergence of costlier treatment options, overall treatment costs declined, possibly due to increased vaccination rates. The study highlights the significant financial strain on the healthcare system and underscores the importance of evidence-based resource allocation to better manage future public health crises.

## 1. Introduction

Coronavirus disease-2019 (COVID-19) caused by severe acute respiratory syndrome coronavirus 2 (SARS-CoV-2) rapidly spread worldwide, registering a total number of infections of more than 700 million [1]. Romania was no exception from the rapid spread of the virus.

**Figure 1:**
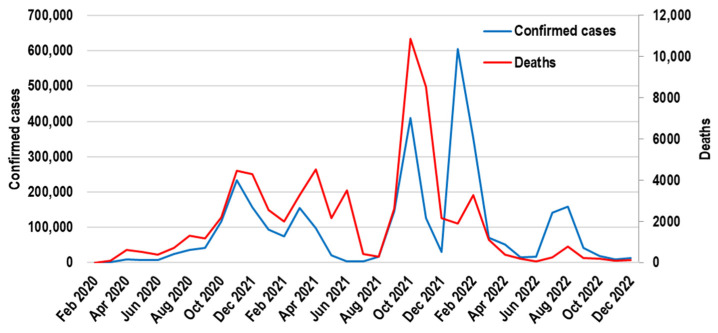
Monthly newly confirmed COVID-19 cases in Romania based on the official data published by the National Institute of Statistics of Romania.

The evolution of the pandemic in Romania is similar to the rest of Europe, registering wave after wave, the largest being reported in January 2022 (Figure 1) [2].

**Figure 2:**
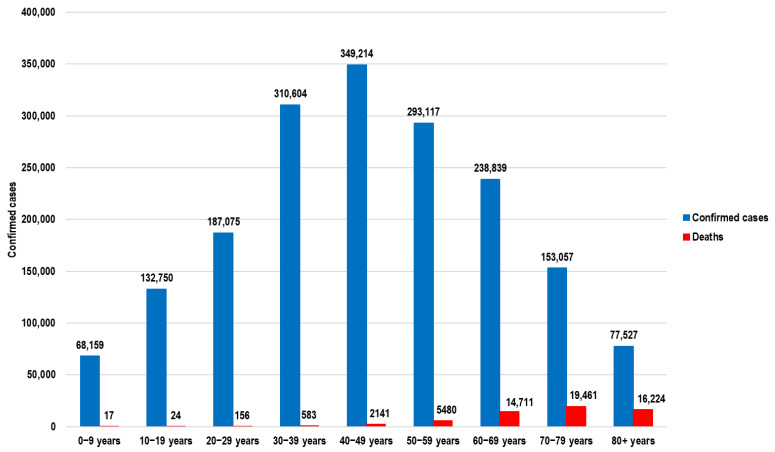
Number of confirmed COVID-19 cases and deaths per age group until Q3 2021 based on the official data published by the National Institute of Statistics of Romania.

Based on the official data, the highest number of cases was reported for the age group of 40–49 (Figure 2) [2] while the average age of the Romanian population in 2019 was 42.1 years [3].

Faced with the pandemic, new problems arose and the existing ones were exacerbated in many fields including national economies (e.g., slowdown of global economic activity) and health systems [4,5]. Ongoing changes in epidemiological guidelines, the introduction of “stay-at-home” policies, medical treatments, hospitalization patterns, and health policies have placed growing financial strain on healthcare systems in many countries [6,7,8].

These evolving factors have directly influenced healthcare resource utilization, including increased average lengths of hospital stays and the adoption of high-cost therapeutic interventions, thereby exacerbating the economic burden associated with the COVID-19 pandemic [7,9]. Assessing all these factors can help decision-makers evaluate their policy-making and develop proper strategic and resource allocation decisions in the future.

Thousands of patients were admitted to hospitals at any given time not only in Europe but all over the world, all translating into costs [1]. A study from Turkey found a direct annual medical cost of purchasing power parities (PPPs) of USD 2.1 billion and a hospital median treatment cost of PPPs of USD 3585.9 [10], in Germany the hospital median treatment costs ranged from EUR 900 to EUR 53,000 per patient [11], while in Greece the mean hospital treatment cost was of about EUR 4347 [12]. Similarly, from the National Health Insurance House (NHIH) perspective, the mean cost per hospitalized COVID-19 patient in the UK was GBP 4847 [13].

Romania continued using the existing Diagnosis-Related Group (DRG) payments for severe respiratory diseases, with adjustments for new ICD-10 codes related to COVID-19 [14]. DRG payments were not changed, but were used to pay for both COVID-19 and non-COVID-19 hospitalizations [14]. New budgets were introduced for hospitals based on pre-agreed contracts, regardless of actual activity levels [14]. Additionally, new budgets were allocated for hiring more personnel to address increased healthcare demands due to COVID-19 [14]. Romanian hospitals were reimbursed for additional running costs, including COVID-19-related expenses such as PPE, hygiene products, and infrastructure adaptation [14].

To this date, only a few studies investigated the direct and indirect economic costs associated with COVID-19, especially in Central and Eastern Europe.

In an attempt to fill in the information gap, we propose an exhaustive approach in order to assess the hospitalization and indirect economic costs of the COVID-19 pandemic in Romania. The main methodological framework used for hospital cost measurement at healthcare (hospital costs) and economic costs were used as earlier defined by C. Jo [15]. A standard controlling methodology was used for hospital cost measurement similar to earlier cost-of-illness studies [16,17].

## 2. Materials and Methods

### 2.1. Data Sources

To estimate the indirect economic costs associated with premature mortality and workdays lost due to COVID-19, data were obtained from official national governmental sources [2,18,19,20] and international databases [21,22]. These sources provided information on the total number of COVID-19 infections, hospital bed-days, and days spent in quarantine or isolation. Additionally, comprehensive inpatient data—including length of hospital stay, number of intensive care unit (ICU) days, diagnostic codes, patient age, and discharge status—were available from a cohort of 60 hospitals.

To evaluate the hospital costs associated with the treatment of COVID-19 patients, detailed economic data were collected from a sample of eight public hospitals. To enhance the generalizability of the analysis, institutions representing varying levels of complexity (two clinical, three county, and three city hospitals) and diverse geographical regions were included. These selection criteria were consistently applied across both costing measurement periods. General hospitalization data were derived from anonymized hospital claims submitted to the National Health Insurance Fund (NHIH), thereby exempting the study from the requirement of ethical committee approval. Costing was conducted using the internal controlling systems implemented within the selected hospitals, the presence of which served as an inclusion criterion for participation [23]. The mean cost per patient episode was calculated for each hospital complexity level. These values were subsequently weighted according to the number of reported patient episodes per level to estimate national-level hospitalization costs.

Given the heightened severity and substantial economic impact of pandemic-related measures during the initial phase—characterized by a state of emergency followed by a state of alert—this study focused on estimating key elements of indirect economic costs incurred between Q2 2020 and Q3 2021. The cost components included in the analysis comprised institutional quarantine costs, economic costs related to premature mortality among COVID-19 patients, and sick leave expenses (for both hospitalized individuals post-discharge and non-hospitalized patients). Additionally, costs associated with the quarantine of confirmed patients’ contacts, parental allowances for supervising children under 12 years during lockdowns and periods of online education, and expenditures related to PCR and rapid antigen testing were considered. Recognizing the temporal variation in hospital costs across Romania during the pandemic, the study also sought to compare hospitalization costs between two distinct periods: Q4 2020–Q3 2021 and Q1 2022–Q4 2022.

The calculation of sick leave costs focused on the working-age population (18–65 years). For these estimations, the national average employment rate from the third quarter of 2021 was used, as it was deemed representative of the Q4 2020–Q3 2021 period based on expert consensus. When multiple approaches for national-level estimation were available, a conservative methodology was adopted by favoring underestimation over overestimation. Estimates of parental allowances for supervising children under 12 years during the lockdown were derived from data provided by the Romanian Court of Accounts’ report on the state of emergency [24]. Government data also included the number of PCR and rapid antigen tests conducted, as well as the approximate number of rapid tests distributed in schools [25]. PCR tests were reimbursed at EUR 41 until 31 March 2021, and at EUR 36 from 1 April to 31 December 2021 [26]. The cost of rapid antigen tests was set at EUR 10.22 for general practitioners. According to expert opinion, these values were below the actual market prices, and such testing costs were not covered by the National Health Insurance House (NHIH). The cost of rapid antigen tests distributed to schools was EUR 2 per test.

### 2.2. Inclusion and Exclusion Criteria

The cases included in the cost analyses were those with at least one specific COVID-19 diagnosis code as defined by the International Statistical Classification of Diseases and Related Health Problems, 10th Revision (ICD-10): U07.1 (COVID-19, virus identified) or U07.2 (COVID-19, virus not identified), or those for which the designated COVID-19 field was completed. Only cases reported to the National Health Insurance House (NHIH) for reimbursement were considered. A single COVID-19 case or hospitalization episode was defined as the period from hospital admission to discharge.

National-level economic cost estimations for the first study interval (Q4 2020–Q3 2021) were based on data obtained from 60 hospitals. The costing analysis focused on the resource utilization and treatment-related expenditures associated with COVID-19 cases managed in the following hospital departments: infectious diseases, pediatric infectious diseases, cardiology, pediatric cardiology, dermatology, palliative care, internal medicine, nephrology, pediatric nephrology, neurology, pediatric neurology, pediatrics, pulmonology, and pediatric pulmonology. These departments were considered to represent the primary settings for COVID-19 patient care. Departments not directly admitting patients for COVID-19—despite incidental positive test results—were excluded from the analysis. Additional exclusion criteria included hospitalizations with diagnostic codes related to acute trauma (e.g., accidents) and cases with a length of stay of zero days, except in instances where the patient had died.

### 2.3. Cost Measurement

In Romania, there is no legal obligation for hospitals to implement controlling systems or to maintain patient-level cost records, except in the case of pharmaceuticals and medication [27]. However, all the hospitals included in this study operated internal controlling systems, which enabled the application of a top-down costing methodology. The cost analysis encompassed direct medical costs (e.g., salary costs of medical and non-medical staff, pharmaceuticals, medical and non-medical supplies, contracted services, and utilities), internal service costs (e.g., laboratory diagnostics, imaging services, pathology, and intensive care unit services), and administrative costs not directly attributable to patient care (e.g., salary costs for administrative personnel, human resources, accounting, statistical, and security staff). In alignment with the study’s conservative approach, expenditures related to investment, amortization, and capital repairs were excluded from the cost calculations.

Hospitalization cost assessments were conducted for two distinct periods: Q4 2020–Q3 2021 and Q1 2022–Q4 2022. This periodization was chosen due to data availability and the consideration that prior to September 2020, hospitalization was mandatory for all COVID-19 cases in Romania, which would have significantly skewed average cost estimations. Outlier cases were included in the analysis and defined as those whose costs exceeded the geometric mean by more than two standard deviations, following methodologies established in prior research [28].

All the cost values originally recorded in Romanian Lei (RON) were converted to Euros (EUR) using the average exchange rates published by the Romanian National Bank: 1 EUR = 4.9011 RON for the first interval and 1 EUR = 4.9315 RON for the second. For economic cost estimations, the average exchange rate over the entire reference period (1 EUR = 4.8906 RON) was applied. The weighted national average cost per hospital and ICU day was determined by calculating the average daily cost for each hospital category and adjusting for the volume of COVID-19 patient discharges per category. This method ensured that variations in discharge volumes across hospitals were appropriately accounted for.

The national average gross monthly salary during the Q2 2020–Q4 2021 period was established at EUR 1141 based on official statistics. Indirect economic cost calculations included an additional 2.25% for the work insurance contribution. Premature mortality costs attributable to COVID-19 were estimated using data from a panel of 60 hospitals, which collectively reported over 13,600 deaths. The analysis focused on fatalities among the working-age population (18–65 years), and total lost active life years were multiplied by the average gross salary to determine associated economic losses.

Data on quarantine and isolation days were available only in an aggregated form, lacking age-specific distribution. To estimate age-specific durations, the distribution of COVID-19 infections by age group was applied to the total, which was further disaggregated using hospital data from the same 60-hospital panel. For institutional quarantine days, hospital days were deducted accordingly to avoid duplication.

Estimates of parental allowances for supervising children under the age of 12 were based on data from the state of emergency period. These figures were then extrapolated to cover the entire relevant timeframe.

### 2.4. Statistical Analysis and Figures

To compare the hospitalization costs and length of stay (LoS) across two distinct periods, as well as among cases of varying complexity (i.e., cases without pneumonia, with pneumonia, admitted to the ICU, and admitted to the ICU with ventilation therapy), the Kruskal–Wallis test was employed. Dunn’s multiple comparisons test was used for selected dataset pairs, while comparisons between the two time periods were analyzed using the Mann–Whitney U test. All the inferential statistical analyses were performed using the GraphPad Prism 5 (GraphPad Software, Boston, MA, USA) with a significance level set at α = 0.05. Figures were generated using Microsoft Excel (Version 2503, Microsoft Corporation, Redmond, WA, USA) and GraphPad Prism 5.

## 3. Results

### 3.1. Hospitalization Cost Measurement

#### 3.1.1. Inpatient Data Analysis

The mean age of the studied cases was 52.06 and 39.17 years for the first (Q4 2020–Q3 2021) and the second intervals (Q1 2021–Q4 2022), respectively. The average length of stay (ALoS) was 8.31 days for the first, and 6.93 days for the second period. ALoS at the ICU was 0.83 days for all the cases and 7.14 days for the cases treated at the ICU for the first interval, and 0.50 days for all the cases and 8.24 days for the cases admitted to the ICU for the second interval (Table 1).

The ALoS both in the general ward (GW) and at the ICU vary according to the hospital complexity level (Table 2).

#### 3.1.2. Cost Data

In the eight included hospitals, 21,607 COVID-19 patient episodes were identified at the included hospital departments, which represent about 3.7% of all the cases reported by hospitals at a national level in the analyzed time interval (the first interval, i.e., Q4 2020–Q3 2021).

**Figure 3:**
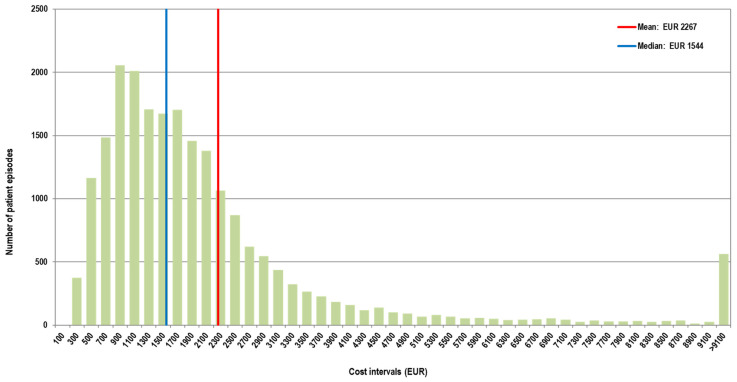
Distribution of cases according to the total cost in the first interval (Q4 2020–Q3 2021). X-axis values represent upper limit of 200 euro cost intervals (bins).

**Figure 4:**
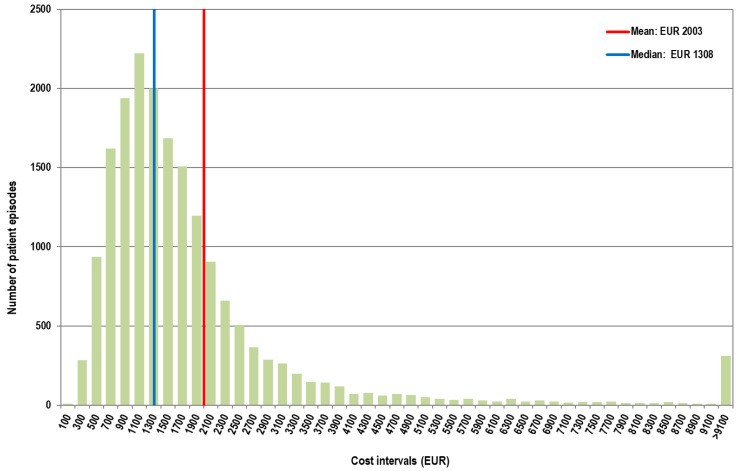
Distribution of cases according to the total cost in the second interval (Q1–Q4 2022). X-axis values represent upper limit of 200 euro cost intervals (bins).

In the first interval, the average cost per patient episode was EUR 2267, while the median was EUR 1544. In the second interval, the average cost per patient episode was EUR 2003, while the median was EUR 1308 (Figure 3 and Figure 4).

The highest mean costs occurred at the clinical-level hospitals (Table 3).

The relatively lengthy hospital stay (compared to the 2019 national average of acute care, of 5.77 days) [22] resulted in the highest registered cost component associated with hospital ward care, followed by the costs of ICU care (Table 4).

**Figure 5:**
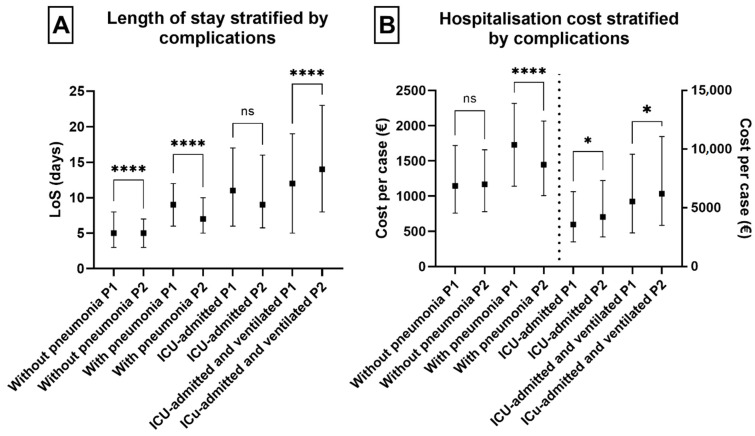
Length of stay and hospitalization costs of cases of different complications for the two studied periods. (**A**): Length of stay (days) stratified by complications; ns: not significant. (**B**): Costs per case stratified by complications (cases that were treated at ICU correspond to the Y axis on the right). Significant differences are noted with * (*p* < 0.05) and **** (*p* < 0.0001), respectively; ns: not significant. P1—first period (Q4 2020–Q3 2021); P2—second period (Q1 2022–Q4 2022).

The level of complication of COVID-19 cases can be assessed through the presence of pneumonia, admission to ICU, and ventilation therapy. The presence of these complications increases the cost. The most resource-intensive care is admission to the ICU with ventilation (Table 5). The statistical differences in length of stay (LoS) between the two periods were highly significant across various complication levels with the exception of non-ventilated ICU cases. Hospitalization costs per patient episode showed no significant difference in cases without pneumonia, highly significant differences in cases with pneumonia, and significant differences in both ventilated and non-ventilated ICU cases (Figure 5).

#### 3.1.3. National Level Hospitalization Costs

The two main cost drivers during hospitalization were hospital ward treatment and ICU treatment. Table 3 presents data on the mean and median cost per patient episode across different hospital levels (clinical, county, and city) over two analyzed periods.

We found that clinical hospitals had the highest mean cost per episode in both periods, EUR 3164 in Q4 2020–Q3 2021, decreasing to EUR 2741 in Q1 2022–Q4 2022.

County hospitals had moderate costs, while city hospitals had the lowest mean cost per episode in both periods: EUR 1832 and EUR 1472, respectively. Across all the hospital levels, costs per episode decreased over time.

Clinical hospitals showed a significant increase in mean cost per day from EUR 342 to EUR 496, suggesting higher per-day treatment expenses despite the decline in total episode costs. County and city hospitals had relatively lower costs per day, with minor variations over time.

For the calculus of weighted average cost/day, the number of cases discharged from hospitals of different complexity levels was taken into consideration. Most hospital days were discharged from city hospitals (38.03%), followed by clinical hospitals (33.49%) and county hospitals (28.49%). Applying these hospital days-based weights to the nationally discharged patient numbers resulted in a national-level weighted average cost of EUR 260/day.

The distribution of patients discharged from the hospitals of various levels shows that most of the patients were treated in city hospitals (37.76%), followed by clinical hospitals (33.29%) and county hospitals (28.95%). Applying these proportions results in a national-level weighted average ICU cost/day of EUR 540 (Table 3).

According to the official data, until the end of 2021, nationally there were a total of 4.69 million COVID-19-related hospital days and 474,713 COVID-19-related ICU days. This yields a total cost of hospitalization of EUR 1.36 billion.

### 3.2. Indirect Economic Costs

#### 3.2.1. National-Level Economic Costs Associated with Hospitalized Patients for the Period Q2 2020–Q3 2021

Aside from hospitalization costs, economic costs are attributed to the patients discharged from the hospital and isolated after the discharge. The total number of days of isolation was 3.55 million, of which 2.50 million days were attributable to the active, 18–65 years age group. Correcting for the employment rate of this age group [3], the resulting number of days was 1.69 million. This, multiplied by the national gross monthly wage [3] and work insurance contributions, resulted in an economic cost of EUR 64.69 million.

#### 3.2.2. Costs Associated with the Premature Death of COVID-19 Cases for the Period Q2 2020–Q3 2021

According to official information, until the end of 2021, in Romania, there were 58,797 deaths attributable to COVID-19. Based on the distribution of deaths per age group (Figure 2), the total number of life years lost for the active population was 164,975 years. Applying the national average gross wage results in a total loss of EUR 2.31 billion.

#### 3.2.3. Costs Associated with the Isolation and Quarantine of Confirmed and Suspected COVID-19 Cases for the Period Q2 2020–Q3 2021

Public information was available regarding the total number of days of quarantine and isolation for the suspected and confirmed COVID-19 cases. The costs associated with the isolation and quarantine of confirmed and suspected COVID-19 cases were calculated, summing up to a total of 52.14 million days. Of these, 36.77 million days occurred in the 18–64 age group. Multiplying that by the employment rate resulted in 24.85 million days. This, multiplied by the national average gross wage, produced a total cost of EUR 951.17 million.

#### 3.2.4. Hotel Costs of Institutional Quarantine for the Period Q2 2020–Q3 2021

Based on official data, 1.06 million days were spent in institutional quarantine, for which the Romanian Government set a tariff of EUR 61 per day. This resulted in a total cost of EUR 64.76 million by the end of 2021.

#### 3.2.5. Parental Allowance for Supervision of Children up to 12 Years for the Period Q2 2020–Q3 2021

The official data reported a cost of EUR 23.07 million for a period of 2 months of state of emergency, during which there were 7 weeks of online school for which parents were eligible for this allowance. Over the total period analyzed, there were 22 weeks (18 weeks in 2020 and 4 weeks in 2021) when parents were eligible. These costs summed up to EUR 72.51 million.

#### 3.2.6. COVID-19 Testing for the Period Q2 2020–Q3 2021

According to government data, until 31.03.2021 a total of 6.74 million PCR COVID-19 tests were performed, and another 4.32 million tests until the end of the year. This results in a total cost of EUR 430.33 million. The number of reported rapid antigen tests was 5.43 million for the analyzed period, summing up to a total of EUR 61.85 million. In schools there were approximately 9 million tests distributed per week, with a total of 4.5 weeks for the analyzed period, resulting in a cost of EUR 82.81 million. The total cost of testing was about EUR 575.00 million based on our conservative approach. This amount does not include the cost of self-testing paid by individuals.

All the cost items including the hospitalization cost, the cost of isolation days for the discharged cases, the costs associated with the premature death of COVID-19 cases, the days of work missed with isolation and quarantine, and the parental allowance for supervision of children sum up to a total cost of EUR 5.39 billion. This represents the total national burden of the COVID-19 pandemic in the first studied interval.

## 4. Discussion

This is the first study in Central and Eastern Europe to estimate the economic costs of the COVID-19 epidemic in monetary terms. We found that besides the very high cost of hospitalization, the indirect economic cost could reach up to 2.5 times the hospitalization cost itself, resulting in a huge economic burden for Romanian society.

The impact of the total cost of EUR 5.39 billion of COVID-19 can be best represented in comparison with the ever-increasing NHIH budget, which in 2019 was EUR 7.99 billion [29]. Considering the number of working adults in Romania, the burden per active person is EUR 639.

It is worth mentioning that health policies and treatment approaches changed greatly over the analyzed period; new treatment options were introduced, and hospitalization policy was changed, as until September 2020 all confirmed cases had to be hospitalized. All these policy changes influenced hospital costs. The costs decreased considerably in the second interval (mean costs per patient episode were EUR 2267 and EUR 2003, respectively). The difference could be caused by a multitude of factors. Surprisingly, the appearance of new and more costly treatment options (i.e., monoclonal antibodies) did not result in higher treatment costs on average, since the costs in the latter studied period were lower than in the previous one. Another factor that possibly influenced the average cost per patient episode was that several hospitals were reconfigured into COVID-19 hospitals. Many wards were closed or barely functioning in these healthcare units due to the lack of patients, nevertheless, the fixed costs remained. Another possible factor could be the age difference between the cases studied in the first and the second interval, with the cases from the second interval being younger, probably with fewer comorbidities and complications. The mean age of the studied cases was lower in the second interval compared to the first. Another factor that might have influenced the costs is the vaccination status of the population since the increasing number of vaccinated subjects in the population might have reduced the number of severely ill patients and/or those in need of mechanical ventilation.

COVID-19 testing in Romania consisted of PCR tests performed in hospitals and testing centers financed by the government and also as out-of-pocket payment. The cost of tests not financed by the government is certainly higher than the financing. Because of this, our calculations underestimate the total cost. The vast majority of rapid antigen tests were financed from private sources, either by individuals or companies. The actual cost, again, is not known, but it is estimated to be higher than the cost set by the government. Aside from this, the population has performed numerous tests not reported officially at home and the cost of these cannot be estimated. The rapid tests distributed to schools were financed by the government. The considerable cost of the distribution of these tests is not included in this study.

Vaccination may have a great effect over the course of a pandemic. At the end of 2021, in Romania, only a relatively small proportion of the population was fully vaccinated (40.5%), whereas the European average was 61.24%, while some European countries, such as Portugal (89%), Spain (81%), Denmark (78%), or Italy (74%), reported much higher percentages [1]. This fact opens opportunities for future studies that could assess the losses due to lack of vaccination or the cost of preventive measures (i.e., vaccination) versus the hospital and societal costs of COVID-19. Nevertheless, it is clear that mitigation strategies evolved since the COVID-19 outbreak and pandemic preparedness as well as health spending are estimated to increase, having a significant impact on the economic burden of the disease, especially by the end of a pandemic period [30]. In such urgent situations, newly introduced health technologies are sometimes procured (vaccines, repurposed medicines, ventilators, etc.) and opportunity costs are ignored due to the time pressure, leading to high costs [31]. This could further increase the burden of the disease.

Our findings are in line with the other results reported in the literature. A 2024 study concluded that the financial impact of COVID-19 hospitalizations in Italy was approximately EUR 3.1 billion in 2020 and EUR 3.6 billion in 2021. The cost per admission was around EUR 8000 for standard care and EUR 24,000 for intensive therapy in both years [32]. The total hospitalization costs (EUR 3.1 billion and EUR 3.6 billion, respectively) are comparable to our findings regarding total hospitalization costs (EUR 1.36 billion).

A 2021 European study reported that the projected hospital admission costs during the first wave of COVID-19 in Europe varied from EUR 25,018 for individuals with type 2 diabetes and well-controlled blood sugar to EUR 57,244 for those with type 1 diabetes and poorly controlled glycemic levels, while the corresponding cost for people without diabetes was estimated at EUR 16,993 [33].

In a 2022 study published by M. Popescu et al., the mean hospital costs of ICU-admitted patient episodes were EUR 10,319 [34]. The median daily total costs in the mentioned paper were EUR 598.4/day. This is comparable to our results (EUR 420 and EUR 600/day for the two studied periods, respectively (Table 3). Despite the higher per-day costs in terms of ICU expenses, the mean and median costs per episode were lower in the second period, possibly due to a lower rate of serious complications. In the first studied period, the number of episodes with ICU-treated patients with ventilation and also those who received mechanical ventilation at ICU were higher (4% and 8% compared to 2% and 4%, respectively) for Q4 2020–Q3 2021 and Q1 2022–Q4 2022 (Table 5). Accordingly, in the earlier mentioned paper by M. Popescu et al., they reported that the degree of lung involvement, need for renal replacement therapy, lower P/F ratio, and treatment with antiviral or immunomodulatory drugs were the factors that correlated strongly with economic outcome (i.e., higher total daily costs) [34]. Herein, we had no access to data regarding all of the mentioned prediction factors, but we consider that these also contributed to our results.

Some limitations of our study are rooted in the quality of the data used in some cases, where data were lacking (e.g., the number of cases for certain days or the number of quarantined or isolated cases), since officially available data on these types of cases were scarce, thus NGOs reports were used as the data source.

To ensure better data quality, verifications were performed from various official sources, and missing data for case numbers were estimated in a conservative manner. Consequently, our findings rather underestimate the total financial costs of COVID-19 in Romania than overestimate it. The exact number of hospitalized COVID-19 patients could not be pinpointed from the available official data, but our confidence in the used proxy data is high.

Medical parameter indicators calculated for the study population, such as the ALoS and the ALoS in ICU, are in line with the findings of studies from other countries [35,36,37].

The figures we present in this paper underestimate the total cost, as we could not include all the relevant cost items like the healthcare costs outside of the hospital setting, including the costs of general practitioners, ambulatory care, one-day hospitalization, and medication outside the hospitals (including over-the-counter drugs), transportation, other out-of-pocket payments (consultations, radiology investigations, rehabilitation services, and medication). Long COVID-19 care and post-COVID-19 rehabilitation were also omitted from this study, costs that also contribute to the total burden of the disease [38]. Similarly, some non-healthcare cost items could not be considered, like the costs of personal caregivers and the loss of businesses during lockdown.

Second, the need for and benefits of post-COVID-19 rehabilitation were proven by various studies [39,40,41,42]. In April 2021, the Romanian Ministry of Health approved the post-COVID-19 recovery protocol, and various hospitals (both public and private hospitals) offer rehabilitation services. The cost of these services was not included in the study, even though they contribute to the pressure on the healthcare system budget.

Third, the mean cost calculated for the pool of the eight hospitals was lower than the costs reported from other countries [10,11,12,13], which would suggest a lower level of hospital costs (mainly labor costs), but also different treatment approaches.

Fourth, reporting the economic costs associated with the premature death of COVID-19 patients is an essential part of the burden of disease studies and it was addressed in several studies involving various countries [43,44], but the comparability of the outcomes is questionable, mostly because of the timeframe measures. Nonetheless, the issue also raises the question of excess mortality. According to international official data, the percentage difference between the cumulative number of deaths since 1 January 2020 and the cumulative projected deaths for the same period based on previous years was 21% for Romania at the end of November 2021 [45].

Generally, societal costs of the COVID-19 pandemic do not involve only the costs incurred with the treatment of confirmed cases and the quarantine of contacts, but also the future (economic and societal) costs of patients who had reduced access to healthcare services during the pandemic. Numerous studies have shown a reduction in healthcare service utilization [46]. The situation is similar in Romania, considering that the number of hospitalizations was reduced by 39.3% in 2020 compared to 2019 and by 38.7% in 2021 compared to 2019, with chronic care being more affected than acute care [47].

Moreover, this study does not consider the collateral economic costs of lockdowns or various restrictions—such as those impacting tourism and industry—even though Romania was among the European countries, alongside Germany, Hungary, and the Netherlands, that implemented an early lockdown [48]. Therefore, the economic impact estimated here could be viewed as an underestimation of the real economic impact induced by COVID-19 on the economy.

Our study has several strengths. First, the strength of this study is the large number of patients included in the study, being the largest study of its kind performed to date in the region. Second, the inclusion of not only healthcare costs but other relevant cost items provides a broader image of the financial and economic pressure brought on by the COVID-19 pandemic. The present report offers valuable insight into the economic impact and the contribution of different cost components to the total costs of the disease. Considering that our results are in line with similar reports from the literature, we consider our results to be highly representative.

## 5. Conclusions

The mean weighted hospital cost per case was EUR 2267. The economic burden of the COVID-19 pandemic reached EUR 5.39 billion. This was calculated until 31 December 2021, but the COVID-19 pandemic is still in progress. The evolution of the costs showed a considerable decrease in the treatment costs despite the appearance of newer, more expensive treatment options. A possible explanation for this apparent contradiction is the increased number of vaccinated among the population.

Our estimation is based on a conservative analysis, suggesting that the actual costs are higher than those assessed, and are projected to increase further. These figures are the results of policy decisions on a national level and put considerable pressure on the healthcare budget.

It is impossible to predict the timing of the outbreak of a new pandemic, but having the results of such studies can support resource allocation decisions in handling future crisis situations.

## Figures and Tables

**Table 1:** Mean value of the medical parameters of the cases studied in the cost estimation.

Factor	Male ^1^		Female ^1^		Total ^1^	
	Q4 2020–Q3 2021	Q1 2022–Q4 2022	Q4 2020–Q3 2021	Q1 2022–Q4 2022	Q4 2020–Q3 2021	Q1 2022–Q4 2022
	(n = 11,316)	(n = 9222)	(n = 10,291)	(n = 8922)	(n = 21,607)	(n = 18,144)
Age (years)	50.58 (49.63–51.53)	36.84 (35.67–38.02)	53.69 (52.75–54.63)	41.58 (40.41–42.76)	52.06 (51.11–53.01)	39.17 (38–40.35)
ALoS (days)	8.27 (8.03–8.5)	6.98 (6.77–7.19)	8.37 (8.15–8.59)	6.89 (6.68–7.09)	8.31 (8.09–8.54)	6.93 (6.73–7.14)
ALoS (ICU) (days) all patient episodes	0.88 (0.75–1.02)	0.54 (0.44–0.65)	0.78 (0.66–0.9)	0.46 (0.35–0.56)	0.83 (0.71–0.96)	0.50 (0.16–0.84)
ALoS (ICU) (days) ICU patient episodes	6.93 (6.63–7.24)	8.15 (7.83–8.46)	7.41 (7.13–7.69)	8.37 (8.01–8.73)	7.14 (6.84–7.43)	8.24 (7.91–8.58)

^1^ mean (95% CI).

**Table 2:** ALoS of the cases studied in the cost estimation according to hospital level.

Hospital Level	ALoS General Ward ^1^		ALoS ICU (All Cases) ^1^		ALoS ICU (Only Cases Treated at the ICU) ^1^	
	Q4 2020–Q3 2021	Q1 2022–Q4 2022	Q4 2020–Q3 2021	Q1 2022–Q4 2022	Q4 2020–Q3 2021	Q1 2022–Q4 2022
Clinical	6.82 (6.64–7.00)	6.14 (5.92–6.35)	1.13 (0.74–1.51)	0.61 (0.22–0.99)	10.56 (10.17–10.94)	10.05 (9.66–10.43)
County	8.05 (7.93–8.18)	7.30 (7.17–7.43)	0.74 (0.52–0.96)	0.41 (0.13–0.69)	5.97 (5.75–6.19)	6.49 (6.24–6.74)
City	6.59 (6.30–6.89)	7.59 (7.29–7.88)	0.68 (0.35–1.02)	0.49 (0.17–0.81)	6.26 (5.93–6.59)	8.16 (7.83–8.48)
Total	8.31 (8.09–8.54)	6.93 (6.73–7.14)	0.83 (0.71–0.96)	0.50 (0.16–0.84)	7.14 (6.84–7.43)	8.24 (7.91–8.58)

^1^ mean (95% CI).

**Table 3:** Mean and median cost/patient episode according to hospital level.

Hospital Level	Studied Patient Episode		Mean Cost/Episode (EUR) (95% CI)		Median Cost/Episode (EUR)		Mean Cost/Day (Except for ICU days) (EUR) (95% CI)		Median Cost/Day (Except for ICU Days) (EUR)		Mean ICU Cost/Day (EUR) (95% CI)		Median ICU Cost/Day (EUR)	
	Q4 2020–Q3 2021	Q1 2022–Q4 2022	Q4 2020– Q3 2021	Q1 2022– Q4 2022	Q4 2020–Q3 2021	Q1 2022–Q4 2022	Q4 2020–Q3 2021	Q1 2022–Q4 2022	Q4 2020–Q3 2021	Q1 2022–Q4 2022	Q4 2020–Q3 2021	Q1 2022–Q4 2022	Q4 2020–Q3 2021	Q1 2022–Q4 2022
Clinical	5791	6688	3164 (2952–3375)	2741 (2424–3058)	1817	1705	342 (189–494)	496 (233–759)	318	388	739 (720–758)	705 (686–724)	370	621
County	12,191	7411	1970 (1900–2040)	1626 (1542–1710)	1504	1142	206 (145–268)	236 (169–303)	197	200	417 (400–434)	607 (600–614)	402	530
City	3625	4045	1832 (1734–1929)	1472 (1400–1543)	1183	982	230 (62–397)	207 (83–332)	185	150	466 (458–475)	422 (418–426)	464	451
Total	21,607	18,144	2267 (2137–2396)	2003 (1799–2207)	1544	1308	243 (128–357)	311 (136–487)	221	230	540 (523–557)	611 (604–617)	420	600

**Table 4:** Cost structure of hospitalized COVID-19 patient episodes.

	Mean ^1^		Median ^1^	
	Q4 2020–Q3 2021	Q1 2022–Q4 2022	Q4 2020–Q3 2021	Q1 2022–Q4 2022
Mean total cost/patient episode	2267 (2137–2396)	2003 (1799–2207)	1544	1308
Hospital ward costs	1160 (1126–1194)	968 (939–996)	988	768
ICU costs ^2^	451 (363–539)	350 (183–516)	0	0
Medication costs	244 (226–261)	164 (113–214)	85	26
Emergency care services costs	87 (84–91)	223 (212–233)	48	53
Diagnostic services costs	156 (149–164)	147 (140–155)	94	103
Other costs	39 (36–42)	30 (27–33)	18	9
Administrative costs	129 (122–136)	121 (109–133)	90	78

^1^ mean (95% CI); ^2^ mean ICU cost (95% CI) calculated per total number of discharged cases (admitted and not admitted to ICU).

**Table 5:** Mean and median cost of COVID-19 cases according to the level of complication.

Level of Complication	Studied Patient Episode Q4 2020–Q3 2021	Studied Patient Episode Q1 2022–Q4 2022	ALoS (Days) Q4 2020–Q3 2021	ALoS (Days) Q1 2022–Q4 2022	Mean Total Cost/Patient Episode (EUR) (95% CI) Q4 2020–Q3 2021	Mean Total Cost/Patient Episode (EUR) (95% CI) Q1 2022–Q4 2022	Median Cost/Patient Episode (EUR) Q4 2020–Q3 2021	Median Cost/Patient Episode (EUR) Q1 2022–Q4 2022
COVID-19 without pneumonia, ventilation, or ICU	10,369	11,292	6.08	5.62	1359 (1322–1396)	1379 (1343–1415)	1147	1168
COVID-19 with pneumonia without ventilation or ICU	8231	5528	9.33	7.88	1838 (1802–1873)	1683 (1654–1712)	1728	1448
COVID-19 treated at the ICU without ventilation	887	434	12.73	11.84	5095 (4908–5281)	5615 (5203–6028)	3582	4229
COVID-19 treated at the ICU with ventilation	1640	668	13.88	16.62	8336 (7979–8694)	12,341 (10,312–14,371)	5546	6210

Of all the cases, 579 were defined as extreme cost cases in the first interval, representing 2.7% of all the analyzed cases, with a mean cost of EUR 18,412 and a median cost of EUR 13,659.

## Data Availability

The datasets generated and analyzed during the present study are not publicly available, but can be provided upon request by contacting László Lorenzovici, Syreon Research Romania, via email at lorenzovici@syreon.ro.
